# Rare allergen sources are clinically relevant sensitizers in Central Europe 

**DOI:** 10.5414/ALX02659E

**Published:** 2026-10-30

**Authors:** Julia Wöhrl, Wolfgang Hemmer, Roland Lang, Katharina Moritz, Felix Wantke, Stefan Wöhrl

**Affiliations:** 1 Floridsdorf Allergy Center (FAZ), Vienna,; 2 Paracelsus Medical University, and; 3 University Clinic of Dermatology and Allergology, Paracelsus Medical University, Salzburg, Austria

**Keywords:** allergen, allergen source, allergy test, atypical, diagnosis, non frequent, infrequent, rare, sensitization, skin prick test

## Abstract

Background: In recent times, diagnosing allergies apart from house dust mite (HDM), birch tree, and grass pollen has become increasingly difficult because allergen manufacturers have withdrawn many commercial skin prick test (SPT) solutions for rare allergen sources. Materials and methods: A cohort of 5,857 patients (mean age 35.9 ± 20.3 years, 60.5% female) was tested with standardized SPT panels comprising 46 inhalant and food allergens at a large allergy outpatient clinic in Vienna. Cases with positive tests and allergen-specific history were judged as “relevant”, those without as “latent”. Results: In the patient cohort, 48.8% presented with a history of allergic rhinoconjunctivitis (ARC), 16.7% with bronchial asthma (BA), 13.2% with atopic dermatitis (AD), and 28.9% with food allergy (FA). Of 113,893 SPTs, 7.0% were relevant, 5.3% were latent, and 87.7% remained negative. The 3 most frequent allergen sources – grass, birch, and HDM – showed the highest ratio of 22.2% relevant compared to 7.1% latent SPTs. The 6 other non-rare allergen sources – European ash tree, mugwort, ragweed, *Alternaria alternata*, cat, and dog – yielded 9.5% positive and 7.4% latent SPT results. The other 34 rare allergen sources resulted in 1.8% positive and 3.9% latent tests. Plantain, horse, hazelnut (food), soymilk, peanut, plane tree, and nettle ranked top among the rare allergens. Among rare allergens, relevant SPTs to horse/rabbit, *Alternaria alternata*, oat flour**,** and rye flour were associated with an increased risk for BA including work-related asthma. Conclusion: 15.4% (1,230/7,985) of all relevant SPTs were observed in “rare allergens”. This demonstrates the necessity of including rare allergens in standard allergy workups.

## Introduction 

Atopic diseases such as allergic rhinoconjunctivitis (ARC), bronchial asthma (BA), atopic dermatitis (AD), and food allergy (FA) are exacerbated by IgE-mediated sensitization to various allergen sources. In vivo skin prick tests (SPTs) are the primary diagnostic measure and used twice as often for the diagnosis of type 1 allergy as the measurement of specific IgE [1]. As one of the major advantages over in vitro tests, SPTs give a result within 15 – 20 minutes, which can be discussed on the spot, sparing the patients a second visit. 

In the past 1.5 decades, many commercial SPT solutions have disappeared from the market in the German-speaking countries Germany, Austria, and Switzerland (often referred to by the umbrella term “D-A-CH”) [2]. In Germany, the number of marketed SPT solutions decreased from 552 to 332 between 2011 and 2022 [3]. In most cases, this was not driven by reasons of marketing authorization but by a cost-cutting management decision. This strategy ignores the niche market of “rare allergens” [4]. However, “rare allergen” is not a scientific but a strategic and political term introduced by the allergen manufacturers. Hence, it is hard to find a scientific definition for a “rare allergen”. The German ordinance on allergen products “Therapieallergene-Verordnung” (therapy allergen-ordinance (TAO)) was introduced in 2008. It lists pollen from grasses (*Poaceae sp.*), birch/hazel/alder trees (*Betula sp*., *Corylus sp.*, *Alnus sp.*), house dust mites (HDMs) (*Dermatophagoides pternonyssinus* and *farinae*), and venom of honeybee (*Apis melifera*) and wasp (*Vespula sp*.) as essential allergen products requiring market authorization [5]. In this strict sense, everything else could be regarded as “rare” and this definition was employed for the German “Seltene-Allergene-Projekt” (“rare allergen project”), where all available diagnostic allergen solutions for SPTs, intradermal tests, and nasal and bronchial provocation tests are listed on the following webpage: https://aeda.de/tabelle-seltene-allergene/ (accessed September 11, 2026; website in German only) [6]. In a broader sense, “rare allergens are defined as allergens from an allergen source recognized by only moderate to low-sized patient populations”, i.e., less than 1 : 100,000 affected citizens [7]. The new Guideline of the European Medicines Agency (EMA) applies the same wording [8]. 

Exposition to environmental allergen sources such as pollen depends heavily on climatic and geographic conditions. What might be rare at one site might be frequent in other areas, and vice versa. In Beijing, China’s capital city, *Artemisia* species (mugwort) constitute the most abundant pollen, while pollen traps in South Africa mainly collect *Poaceae* (grass) followed by *Cupressus* (cypress tree) pollen [9, 10]. Hence, from a global perspective, there cannot be a uniform definition for “rare” or “frequent” allergens. 

Also, IgE-mediated FA is neglected in this context. Few would consider prominent food allergens such as peanut (*Arachis hypogaea*), hazelnut (*Coryllus avellana*), cow’s milk (*Bos domesticus*), or hen’s egg (*Gallus domesticus*) to be truly “rare”, which – however – is the case. Additionally, there are many more allergenic foods. The WHO/IUIS allergen database lists 412 molecular food allergens (http://allergen.org/; accessed September 11, 2026) and the one maintained by the University of Nebraska 794 (http://www.allergenonline.org/; accessed September 11, 2026). Many of these food allergens have been observed only in small case series. Hoffmann-Sommergruber [11] tried to explain how commonly consumed foods can cause FAs in only a minority of individuals making them “rare”:

- Allergens present in exotic foods may have been introduced only recently into new geographic regions, leaving insufficient time for sensitization to occur in a substantial proportion of the population.
- Low-allergenic food allergens will always sensitize only a very small proportion of the population.
- Food processing can down- or upregulate the immunological potential of known food allergens: e.g., heating denatures Bet v 1 and other PR10 proteins, which reduces birch-pollen-related cross-reactions to foods such as apple (*Malus domestica* Mal d 1) or hazelnut (*Corylus avellana* Cor a 1), etc.; In contrast, roasting increases allergenicity of the clinically most relevant 2S albumins in roasted nuts and storage fruits such as peanut (*Arachis hypogaea* Ara h 2 and 6), hazelnut (Cor a 14), or sesame (*Sesamum indicum* Ses i 1 and 2), etc. from weak to strong.
- Rare allergens can also be defined by a very low content in normal food so that only highly allergic patients will react.

Annex I of the European CMDh/399/2019 defines the following inhalant and food allergen sources as frequent: pollen of sweet grasses (*Poaceae*), the birch tree group, olive/ash trees (*Oleaceae*), cypress (*Cupressaceae*), ragweed (*Ambrosia artemisiifolia*), pellitory (*Parietaria judaica*), HDMs of the *Dermatophagoides* genus, honeybee (*Apis mellifera*) and wasp (*Vespula sp.*) venom, cat (*Felis domestica*), peanut, and peach (*Prunus persica*) [12]. In this sense, all other allergen sources could be defined as rare. 

One of the reasons behind the “rare allergen” issue is likely that the allergen manufacturers usually cannot sell enough allergen immunotherapy (AIT) preparations for allergens sources other than the most common ones. This is particularly relevant to food allergens, for which no AIT products are currently commercially available. Therefore, from a commercial point of view, it is tempting to categorize all allergens other than the frequent ones that represent a significant market share as “rare”. The immediate past president of the allergy specialists at the European Medical Chamber in Bruxelles (UEMS), Norbert Mülleneisen [13], reported that only 0.7% of AITs in Germany were conducted with “rare allergen” sources not listed in the TAO. 

Is it justified to focus only on the most frequent allergen sources? Studies in the past have already demonstrated that allergic patients are sensitized to many more allergen sources (e.g., to ragweed/*Ambrosia sp.*) [14]. Höflich et al. [15] also described plane tree (*Platanus orientalis*), *Ambrosia* and *Artemisia* as important allergen sources on what they call “non frequent” pollen allergens in the two German federal states Bavaria and North Rhine Westphalia. Mühlheimer et al. [16] define the allergen sources ash tree pollen (*Fraxinus excelsior*), all weed pollen, storage mites (*Tyrophagus sp*., *Acarus sp*., *Lepidoglyphus sp*.), pet animals apart from cat and all molds as “rare”, and we used their clinical and pragmatic definitions for our manuscript with minor modifications based on local conditions (see Definitions in Materials and methods section). 

In this study, we investigated how many patients are sensitized to the so-called “rare” allergens in Eastern Austria/Central Europe with partly Alpine and partly Pannonic climatic conditions. As a secondary aim, we studied the clinical relevance of these sensitizations. We used a previously published dataset of 5,857 patients tested in 2018 at a time when a broad spectrum of 46 commercial SPT solutions to inhalant and food allergen sources was still available [17, 18]. The clinical relevance was categorized into “relevant” sensitizations in patients with and into “latent” in those without corresponding symptoms to the positively tested allergen source. The influence of the underlying atopic disease on the sensitization patterns was studied as a tertiary outcome. 

## Materials and methods 

### Patients 

We analyzed unreported parts of a previously published dataset [17, 18]. In short, there was a cohort of 5,857 routine patients seen by SW in 2018 at the Floridsdorf Allergy Center (FAZ). We had recorded age, gender, underlying atopic disease, wheal-size, and the allergen-specific history concerning relevance for all positive SPTs. The clinical relevance (“relevant” or “latent”) was judged on a clinical basis by specifically interrogating the patient concerning allergen-specific symptoms for each SPT with a positive result. Specific IgE to the major, molecular allergens Der p1/2, Phl p1/5, Bet v1, Fel d1, Ara h2, cow’s milk extract, or hen’s egg extract was determined together with total IgE, and in the case of anaphylaxis, tryptase, which had already been published elsewhere [18]. 

The basic information including the underlying atopic disease is depicted in Table 1. 50.2% patients (2,941/5,857) had completely negative SPTs. 49.8% (n = 2,916) had at least 1 positive SPT supporting one of the four atopic diagnoses: ARC, BA, AD, or FA; 11.2% (654) suffered from more than one atopic condition and only 1 patient (0.017%) was suffering from all of them. 

### Skin prick tests 

The SPT solutions to common and rare allergens were bought from ALK-Abelló (Hørsholm, Denmark) and Bencard/Allergy Therapeutics (Worthing, UK) and were used according to availability. The available SPT solutions at the time of the 2018 study are listed under their package name in Table 2. 37.0% (17 / 46) of the commercially available, standardized “rare” SPT solutions used in this study had already been withdrawn from the market at the time of writing this manuscript in 2026 and are highlighted in pink. 

All patients were submitted to one of the four types of standard series of SPTs, depending on their reported symptoms (inhalant, food, mixture of inhalant/food for adults, mixture of inhalant/food for kids). Each series could be expanded according to specific information in the patient’s history. Overall, there were 113,893 single SPTs (Supplemental Table 1). 

The three inhalant allergens HDM, *Betula*, and *Artemisia* had been included into the FA panel as they can produce typical cross-reactions with food allergens: the so-called secondary or type B FAs: *Betula* (Bet v1 PR10 protein) ↔ soy protein (*Glycine max* Gly m4) ↔ nuts (e.g. hazelnut Cor a1) ↔ stone fruit (e.g. apple Mal d1); *Artemisia mugwort *(ns LTP Art v3) ↔ peach (Pru p3); HDM (tropomyosin Der p10) ↔ shrimp (*Penaeus monodon* Pen m1); HDM, *Betula*, and *Artemisia* pollen had therefore been included in the food allergen series. 

### Definitions: frequent, non-rare, rare 

We used the following three categories (Table 2). 

**Frequent allergen sources**

Six frequent allergens belonging to three groups were defined as frequent according to the German guideline [5]:

- *Poaceae* (commercial names “6 grasses-mix” and “ryegrass”)
- *Betulaceae* (commercial names “3 trees-mix” and “hazel pollen”)
- HDM (commercial names “house dust mite I” and “house dust mite II”, also known as “European” and “American House Dust Mite”, also known as *Dermatophagoides pteronyssinus* or *farinae*, respectively).

Due to their importance, all these three allergens were tested with two different, but cross-reactive SPT solutions as listed in Table 2. In nearly 100% of the cases, both SPTs gave the same result. This can be explained by sequence homologies of the shared major allergens of more than 70%: Poaceae: Phl p1 ↔ rye grass *Lollium perenne* Lol p1/Phl p5 ↔ Lol p5; *Betula*: Bet v1 ↔ Cor a1 ↔ Aln g1; HDM: Der p1 ↔ Der f1/Der p2 ↔ Der f2/Der p23 ↔ Der f23, respectively. All Latin and trivial names of the study allergens are listed in column 2 – 4 of Supplemental Table 1. 

**Non-rare allergen sources**

We categorized the three allergen sources as defined in the publication by Höflich et al. [15] *Fraxinus*, *Artemisia*, and *Ambrosia* as non-rare, but due to the high number of positive tests in this Austrian cohort we expanded this category to the spores of the mold *Alternaria alternata* and the dander of the furry pet animals cat (*Felis domestica*) and dog (*Canis familiaris*) to finally end up with a number of six non-rare allergen sources. 

**Rare allergen sources**

The remaining 34 allergen sources, among them all food allergens, and other high-risk allergen sources such as Hevea brasiliensis latex, were categorized as “rare”. 

### Statistics 

MedCalc Statistical Software version 23.5.2 (MedCalc Software Ltd, Ostend, Belgium; https://www.medcalc.org; 2026) was used for statistical analysis. For analysis of 2 × 2 categorial data, we employed a two-sided χ^2^-test. For analyzing larger crosstabs, we used a modified variant, the “χ^2^-test for trend” also known as “Cochran–Armitage test for trend”. A p-value of < 0.05 was considered significant. 

The figures were produced using Microsoft Excel for Windows Version 2603. As described in the Section “Frequent allergen sources” the results of SPTs with “grass mix” and “rye pollen” were subsumed as “*Poaceae*”, “birch trees mix” and “hazel pollen” as “*Betula*”, and *Dermatophagoides pteronyssinus* and *Dermatophagoides farinae* as “house dust mites HDM”. 

## Results 

There were 2,856 (48.8%) patients suffering from ARC as their referral diagnosis, 981 (16.7%) with asthma (Table 1). Additionally, 1,695 (28.9%) had suspected FA and 775 (13.2%) had AD; The majority (60.5%) were female and the mean age was 35.9 ± 20.3 years. 

Supplemental Table 1 summarizes the complete data set. There were 113,893 single SPTs, grouped in four different panels as described in “Materials and methods”. Of these, 7,985 (7.0%) were relevant and 6,008 (5.3%) latent, yielding 13,993 (12.3%) positive and 99,900 negative tests (87.7%). Disregarding the information for clinical relevance, positive SPTs were highest for frequent allergen sources, intermediate for non-rare allergens, and lowest for rare allergens (22.2 vs. 9.5 vs. 1.8%, p < 0.0001, χ^2^-test). Likewise, the ratio of relevant-to-latent SPTs declined along these categories (3.1, 1.3, 0.5, p < 0.0001, χ^2^-test). Summing up this first part, frequent allergen sources yielded both the highest positivity rates and were of greatest clinical relevance. 

Positive SPTs exceeding 5% were observed for the top 3 and the non-rare allergens (Supplemental Table 1). Notably, even allergens classified as rare, including Plantago pollen, horse, hazelnut (food), walnut (food), soymilk, shrimp, *Tyrophagus*, rabbit, *Acarus* and *Lepdioglyphus*, rye flour, and bird feather mix showed high positivity rates. However, walnut relied only on the small number of 4 positive tests and the results must therefore be interpreted with caution (Supplemental Table 1). Overall, these results suggest that allergens categorized as “rare” may be clinically more relevant than previously thought. 

Figure 1 illustrates allergen- and disease-specific clinical relevance, displayed as the ratio of relevant (red) to latent (orange) SPTs. In ARC, *Poaceae*, *Betula*, and HDM were the most frequent and relevant allergens (Figure 1a), while *Fraxinus*, cat, *Artemisia*, *Ambrosia*, *Alternaria*, rabbit, dog, and shrimp, which is partly cross-reactive with HDM, were also relevant in > 50% of the cases. In BA, HDM and *Betula* changed rank (Figure 1b), the furry animals cat, dog, and horse as well as *Alternaria* became more relevant. Surprisingly, the food allergens rye flour, peach/LTP, shrimp and *Tyrophagus*, *Acarus*, and *Lepidoglyphus* emerged as relevant. In AD, the overall relevance was lower (Figure 1c) although 7 inhalant allergen sources still exceeded 50%. Due to AD’s much closer association with FA, cow’s milk and hen’s egg were rare but relevant allergens. 

Contrasting the peanut-dominated Anglo-Saxon populations, in our adult-dominated Austrian cohort, hazelnut and walnut were the most relevant food allergens, while peanut ranked 7^th^ with a lower clinical relevance (Figure 1d). *Artemisia* with its cross-reactivity to food ranked 6^th^ followed by shrimp, soy, and peach/LTP. Hen’s egg and cow’s milk were also relevant but less prominently, while wheat and oat flour contributed only marginally as food allergens. Confirming experiences from other landlocked countries, fish allergy was rare in our cohort. 

Figure 2 summarizes the distribution of positive SPTs according to the underlying atopic disease. Significant differences between the inhalant diseases ARC/BA and AD/FA were observed only for *Poaceae*, *Betula*, and HDM (p < 0.0001, χ^2^-test) (Figure 2a). Hazelnut dominated in FA compared to the other three atopic conditions (p < 0.0001, χ^2^-test) (Figure 2b). Unexpectedly, rye flour and walnut were associated rather with BA than with FA (p < 0.0001, χ^2^-test). Again, the findings for walnut should be taken with caution because of the small number of 3 positive out of 13 tests. 

Figure 3 illustrates how many clinically relevant sensitizations would have been lost in ARC if rare allergens had been omitted in the test series. Frequent allergen sources accounted for 43.8% of relevant SPTs, none-rare allergens for 24.3%, and rare allergens for 31.9%! 

## Discussion 

Patients are often poly-sensitized to a variety of allergen sources. Both, SPT and measurement of specific IgE are screening tools for finding the elicitors of IgE-mediated diseases. SPTs offer the benefit of few technical prerequisites apart from a refrigerator for the cooling of the extracts and a skilled nurse or technician for performing the tests. 

But what happens if the patient has a false negative test? There are several reasons explaining such a situation. The sensitivity of the test system could be too low. Alternatively, the patient might be sensitized to a rare allergen not included in standard test panels. Our study showed that SPTs with rare and unexpected allergens picked up clinically relevant sensitizations in nearly a third of the patients. Multiplex sIgE panels such as ISAC or ALEX also include rarer allergen sources. Höflich et al. [15] used SPTs and ISAC in their study under climatic conditions similar to those in Eastern Austria and showed that *Fraxinus* (ash tree pollen) and the highly cross-reactive *Olea europea* (olive tree pollen) ranked 4^th^ like in our results. Thus, omitting rare allergens would have led to a false negative allergy diagnosis in 1/3 of the cases. This finding challenges the designation of such allergens as “rare”. 

Typically, Central and North-Western European pollen monitoring does not include the more complex, Mediterranean pollen sources. But the recent changes in climatic conditions might have already shifted the Central European situation to a more Mediterranean pattern because *Plantago*, *Platanus sp.* (plane tree), *Urtica sp*.(nettle), *Atriplex sp*. (saltbush), and the mold *Cladosporium sp*. ranked top among the rare allergen sources. Hence, these allergen sources should be included into standard allergy screening and pollen monitoring in Central and North-Western Europe. 

How many tests are required for routine testing? The principle of cross-reactivity between homologous allergens in major allergen groups has already been introduced to reduce the number required for testing [19]. E.g., the beech trees *Betula/Alnus* (alder)/*Corylus*/*Quercus* (oak) share a very similar major allergen from the PR10 family: Bet v1/Aln g1/Cor a1/Que c1; hence, testing one representant may be good enough for the whole group. The same is true for the hundreds of sweet grasses (*Poaceae*) and the various *Dermatophagoides* species. Hence, we merged the two SPTs for each of these three TAO allergen groups and did not analyze them separately as six but as three allergen groups. However, this principle cannot be automatically applied to the other non-rare and rare allergens. In Austria, the pollen seasons of the *Asteraceae* can be separated by history with *Artemisia* pollen peaks in August (major allergen Art v1) and later Ambrosia (major allergen Amb a1) from September to October. Some of the *Artemisia*/*Ambrosia* co-sensitizations can be explained by a true cross-reactivity from *Ambrosia* Amb a1 to Art v6 from *Artemisia* [20] and also vice-versa from Art v1 to Amb a4 [21]. 

Contradicting data from warmer, tropical climates, cockroach (*Blatella germanica*) and the most important tropical mite *Blomia sp.* did not play a significant role in Central and North-Western European climatic and housing conditions [22]. This was different for *Lepidoglyphus*, *Tyrophagus*, and *Acarus* that showed a trend, though not significant, towards higher sensitization rates in asthma, which had been described by Langley et al. [23] a long time ago. Among the molds, *Alternaria* was so frequent that we classified it as non-rare allergen, while all other molds, among them *Cladosporium*, only played a minor role, which has remained unchanged since our last analysis published in 2009 [14]. 

Pet animals are another important group of allergen sources. Cat/dog were among the most frequent among the non-rare and horse/rabbit among the rare allergen sources. The clinical relevance was very high and peaked for rabbit in asthmatic subjects (Figure 1c). In an earlier study, we had already demonstrated that cat allergy with sensitization to Fel d1 often happened in patients not owning a cat while most other pet-allergic patients were keeping furry animals [24]. 

The four atopic diseases ARC/BA/AD/FA are clearly connected. AD and FA mark the beginning of the development in small children, formerly coined as “atopic march” [25]. Although in recent times this concept was dismissed, it still is clear that preschool children display a sensitization pattern completely different from that of schoolchildren. This has already been analyzed and published by us before [17]. Primary food allergens especially peanut, hen’s egg, and cow’s milk had dominated over inhalation allergen sources in preschoolers. This pattern reverted from the age of 6 when the adult pattern with inhalation allergens began to dominate over FA. If we want to achieve change, we need to address these very young patient groups, as Eiwegger and Pfaar [26] recently outlined in an editorial. 

At a first glance, the high prevalence and clinical relevance of hazelnut/walnut with an at the same time lower relevance of peanut was a bit surprising. This was in line with recently published data showing that Western Europe is a special global region where hazelnut dominates over peanut allergy [27]. As there had not been any commercially available SPT solution for cashew, we could not evaluate this 4^th^ important, Central and Western European nut allergen [28]. 

Contradicting common beliefs, IgE in AD patients was not only directed against food allergens in toddlers and infants but also against aeroallergens in adults. This clinical observation is often ignored in adults, although Werfel et al. [29] had already triggered AD exacerbations by exposing AD patients to *Poaceae* pollen in experimental pollen chamber challenges. From a dermatological perspective, the clinical importance of allergy in AD patients is often neglected, e.g., the living update of the European atopic eczema guideline does not even list allergy testing among the recommended diagnostic measures [30]. 

In a previous study, we compared the sensitivity of SPTs with molecular sIgE testing to the major allergen sources of *Betula*, *Poaceae* pollen, HDM, and cat. We found that extract-based SPTs are far more sensitive than the modern molecular tests against the major allergens Bet v1/Phl p1 & 5/Der p 1 & 2/Fel d1 in 15% up to 28% of the patients [18]. We concluded that at the time being, molecular in vitro tests cannot substitute extract-based testing due to their limited sensitivity. 

Unfortunately, by the end of this study in 2026, 50% (17/34) of the commercial SPTs solutions for rare allergens that were still available in 2018 had already been withdrawn from the market so that this study would not be possible today (Table 2). Some clinical research groups have already tried to produce their own food extracts like in ancient times of allergology [31]. In most cases, non-standardized prick-to-prick tests with fresh or frozen foods are sufficient for everyday clinical practice, but the preparation is much more time-consuming than extract-based testing and not standardized [32]. 

The huge number of patients and the high number of available, standardized SPT solutions back in 2018 constituted the major strengths of this study. Another advantage over most other studies of this kind was the subclassification of positive SPTs into, on the one hand, clinically relevant allergies and, on the other hand, clinically irrelevant, latent sensitizations. However, this could be also regarded as a disadvantage, as the clinical classification by an experienced allergologist did not rely on objective parameters such as provocation tests. However, concerning the vast number of SPTs of more than 100,000, provocation tests would have largely overstretched the resources available for this routine data set. Due to limited resources for routine patients, sIgE had only be determined against the major allergens from the frequent allergen sources Betula, Poaceae, and HDM, which had been described before [18]. Positive SPTs to all other allergen sources had only been backed up by specific IgE determination in a non-systematic approach so that the data were far too heterogenous to analyze them for this study, which is one of its’ major drawbacks. 

## Conclusion 

Sensitization to rare allergen sources was found in one third of the patients. Without testing for rare allergens, clinically relevant sensitizations would have been missed. Although frequent and non-rare allergens were clinically more often relevant than rare allergens, sensitizations to rare allergens demonstrated clinical relevance in up to 1.8% of all tests. This was higher than expected. Rare allergens need to be included in standard allergy test panels. 

## Acknowledgment 

Benjamin Heindl, Tobias Gurecny (formerly Braunsteiner), and Livia Klug had established the database during their medical diploma theses. The analysis of unused parts of their database is thankfully acknowledged. This paper presents Julia Wöhrl’s medical bachelor’s thesis from the Paracelsus Private Medical University in Salzburg, Austria. 

## Ethics approval 

The construction and analysis of the database were approved by three ethical approvals of the Medical University of Vienna: #2213/2018, #2212/2018, #1152/2019. 

## Authors‘ contributions 

JW: retrieved and analyzed the data. WH: co-analyzed the data and revised the manuscript. RL: supervised JW’s medical bachelor thesis. KM & FW: supported in preparing the manuscript. SW: conceived the study and drafted the manuscript. 

## Funding 

This investigator study was self funded by the FAZ. 

## Conflict of interest 

SW: received speaker fees from ALK-Abelló, Bencard, and ThermoFisherScientific; all other authors declare no conflicts of interest. 

**Table 1.** Basic characteristics of the patients (n = 5,857)

**Age (a)**	**Female (n)**	**Male (n)**	**Allergic rhinoconjunctivitis (n)**	**Asthma (n)**	**Atopic dermatitis (n)**	**Food allergy (n)**
35.9 ± SD 20.3	3,544	2,313	2,856	981	775	1,695
	60.5%	39.5%	48.8%	16.7%	13.2%	28.9%

**Table 2.** Commercially available skin prick test solutions used in this study. The allergens are named according to the manufacturer‘s commercial product code.

**Exposure**	**Trivial name**
Inhalative	House dust mite I
Inhalative	House dust mite II
Inhalative	Acarus siro
Inhalative	Lepidoglyphus
Inhalative	Tyrophagus
Inhalative	**Blomia**
Inhalative	**Cockroach**
Food	Shrimp
Inhalative	**Latex**
Inhalative	Cat
Inhalative	Dog
Inhalative	**Horse**
Inhalative	**Rabbit**
Inhalative	**Bird-feather-Mix**
Inhalative	Hazelpollen
Inhalative	Birch-Tree-Mix
Inhalative	Ash-tree
Inhalative	Plane tree
Inhalative	Grass-Mix
Inhalative	Rye-Pollen
Inhalative	Plantain
Inhalative	Mugwort
Inhalative	Ragweed
Inhalative	**Pellitory**
Inhalative	Saltbush
Inhalative	**Nettle**
Inhalative	Alternaria
Inhalative	Cladosporium
Inhalative	**Penicillium**
Inhalative	Aspergillus
Inhalative	Date Palm (Profilin)
Food	Peach (LTP)
Food	**Walnut (food)**
Food	**Hazelnut (food)**
Food	Peanut
Food	Soybean
Food	**Soymilk**
Food	**Oat flour**
Food	Rye flour
Food	Wheat flour
Food	**Pork**
Food	**Chicken meat**
Food	**Eggwhite**
Food	**Eggyolk**
Food	Cod Fish
Food	Cow’s milk

**Definitions in this study**	
Frequent	
Non-rare	
Rare	
**No commercial skin test solution available in Austria by 2026**	

**Figure 1:**
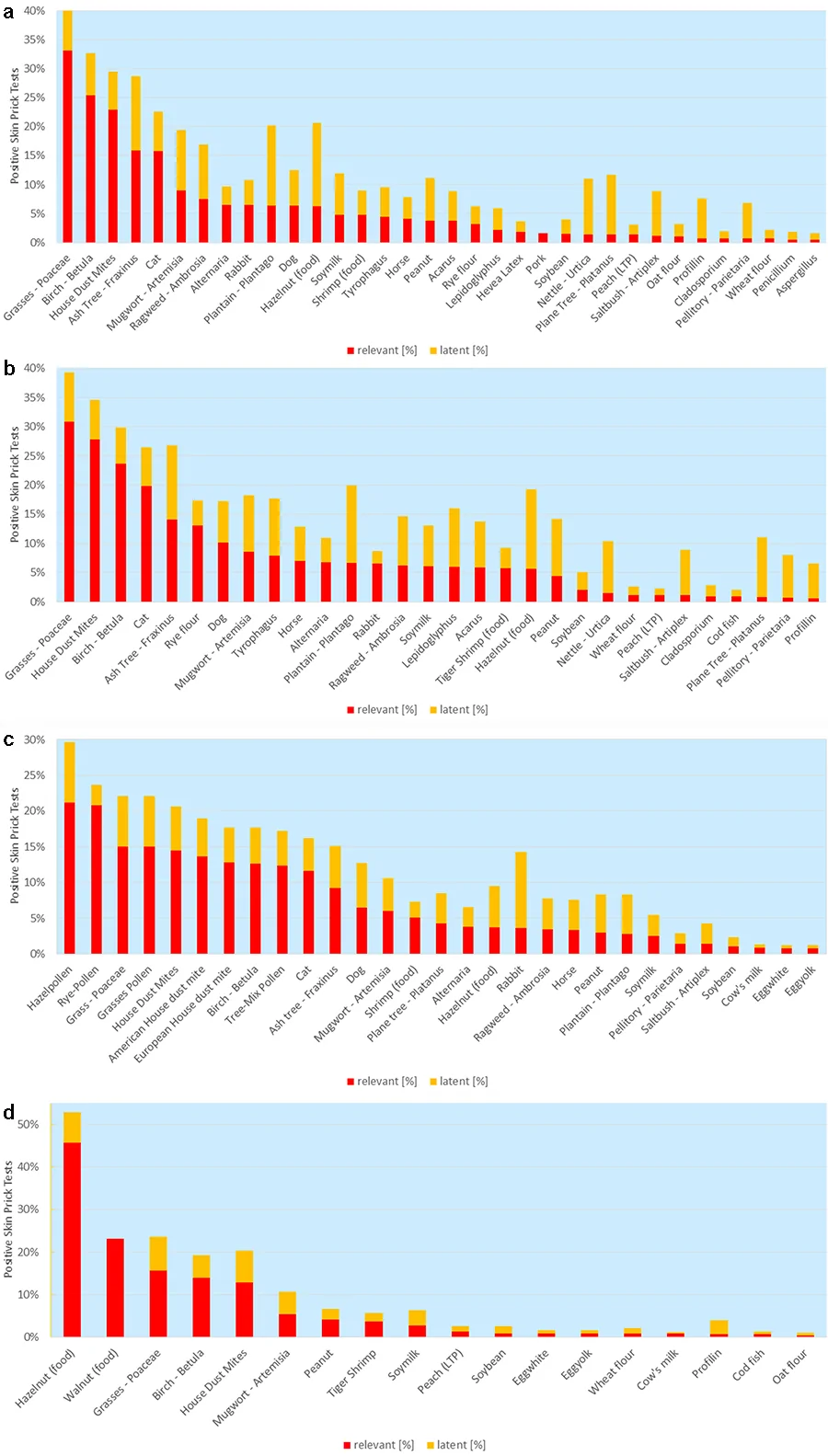
Positive skin prick tests. a: In 2,856 allergic rhinoconjunctivitis patients, cutoff ≥ 5 test results. b: In 981 bronchial asthma patients, cutoff ≥ 3. c: In 775 atopic dermatitis, cutoff ≥ 5. d: In 1,695 patients with suspected food allergy, cutoff ≥ 2. Results sorted according to positive, relevant test rates.

**Figure 2:**
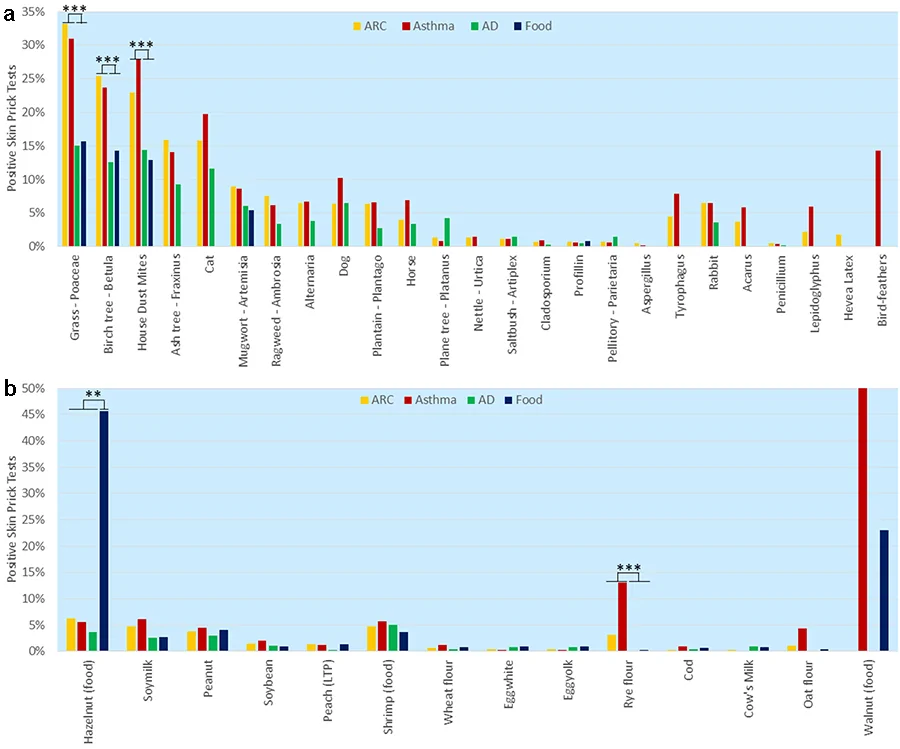
Positive, clinically relevant skin prick tests depending upon the underlying atopic disease (%). AD = atopic dermatitis; ARC = allergic rhinoconjunctivitis. a: Inhalant allergen sources. b: Food allergen sources. **Significant difference between food allergy and the three other atopic diseases, χ^2^ test. ***Highly significant difference between inhalant and non-inhalant allergens, χ^2^ test.

**Figure 3:**
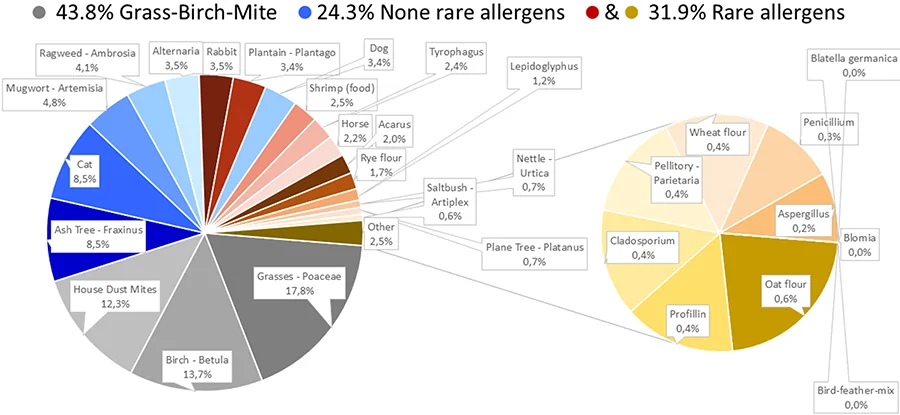
What would be missed when omitting rare allergens? Skin prick tests in 2,856 allergic rhinoconjunctivitis patients: 43.8% frequent allergens: grass-tree-mite (grey), 24.3% none-rare allergens (blue), 31.9% rare allergens (red and brown).

### Supplemental material

Supplemental material Supplemental table
